# Single-molecule tracking (SMT): a window into live-cell transcription biochemistry

**DOI:** 10.1042/BST20221242

**Published:** 2023-03-06

**Authors:** Liza Dahal, Nike Walther, Robert Tjian, Xavier Darzacq, Thomas G.W. Graham

**Affiliations:** 1 Department of Molecular and Cell Biology, University of California, Berkeley, Berkeley, U.S.A.; 2 Howard Hughes Medical Institute, University of California, Berkeley, Berkeley, U.S.A.; 3 Li Ka Shing Center for Biomedical & Health Sciences, University of California, Berkeley, Berkeley, U.S.A.

**Keywords:** live cell biochemistry, molecular interactions, proximity assisted activation, single molecule tracking, transcription

## Abstract

How molecules interact governs how they move. Single-molecule tracking (SMT) thus provides a unique window into the dynamic interactions of biomolecules within live cells. Using transcription regulation as a case study, we describe how SMT works, what it can tell us about molecular biology, and how it has changed our perspective on the inner workings of the nucleus. We also describe what SMT cannot yet tell us and how new technical advances seek to overcome its limitations. This ongoing progress will be imperative to address outstanding questions about how dynamic molecular machines function in live cells.

## Introduction

An increasing number of labs around the world deploy various methods to track single endogenous protein molecules in cells, tissues, and even whole organisms to probe their biochemical behavior in their native environment. Here we will discuss how single-molecule tracking (SMT) is performed, what we can learn from it, and how ongoing technical advances are making SMT more informative. SMT has diverse applications in cell and molecular biology, from studying the translocation of molecular motors along the cytoskeleton to monitoring interactions of membrane receptors. Each biological problem poses its own distinct challenges and requires different methodologies [1–3]. In this review, we will cover two specific applications of SMT: fast tracking of diffusing molecules and slower tracking of bound molecules in the nucleus. These methods, which require specialized analysis tools, enable a new form of ‘live-cell biochemistry’ that can distinguish complexes and measure kinetics. Here, we will focus on applications in the field of transcription regulation and discuss a few selected examples.

## Transcription regulation as a case study: what do we want to understand?

Switching a gene on or off involves multitudinous interactions between hundreds of proteins. Protein-encoding genes are transcribed by RNA polymerase II (Pol II), which initiates transcription following assembly of general transcription factors (GTFs) into a pre-initiation complex (PIC) at the gene promoter [4,5]. Transcription initiation by Pol II and release from promoter-proximal pausing are tightly regulated by sequence-specific transcription factors (TFs), which bind short DNA sequences either adjacent to the transcription start site or thousands of base pairs away within enhancers [6]. TFs in turn bind an assortment of proteins and protein complexes called coactivators and corepressors, that activate or repress transcription through diverse mechanisms [7–9]. A critical and outstanding challenge in molecular biology is to understand how this vast network of molecular interactions gives rise to precise patterns of gene expression [10–12]. Making progress toward this goal will require developing new ways to measure protein–DNA and protein–protein interactions (PPIs) of TFs, GTFs, and cofactors in live cells. While SMT has provided a new window into these interactions, its practitioners are still learning to peer through that window and decipher what we see.

## How SMT works

**Figure 1.**
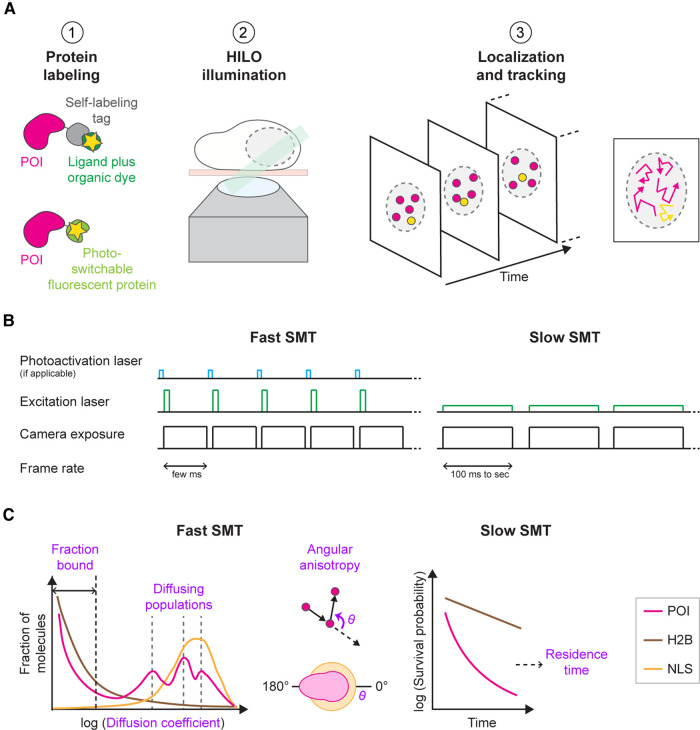
Schematic of live-cell SMT methodology and measurements. (**A**) Typical workflow for SMT of tagged nuclear-localizing TFs. Step 1: The protein of interest (POI, magenta) is tagged either with a self-labeling tag (e.g. HaloTag, gray) and sparsely labeled with a tag-specific ligand (dark green) coupled to a bright and photostable organic dye (yellow star; top) or with a photoswitchable fluorescent protein (light green with yellow star; bottom). Step 2: Cells growing in a monolayer on top of a glass coverslip (beige) are imaged by HILO illumination (green) to visualize single molecules in the nucleus (round dashed line) over time. Step 3: Single molecules (magenta, yellow) are then localized in each frame of a single-molecule movie and tracked over consecutive frames (left), resulting in single-molecule trajectories (right). (**B**) Illumination schemes for fast (left) and slow SMT (right). Fast SMT employs short camera frame rates of only a few milliseconds (black) combined with short stroboscopic excitation pulses at high laser power at the beginning of each frame (green). If required, short photoactivation pulses (blue) are placed during the camera dead time between frames. Slow SMT uses longer frame rates of hundreds of milliseconds to seconds (black), combined with continuous excitation at low laser power (green), to visualize bound molecules. (**C**) Data analysis of single-molecule trajectories. Fast SMT (left) provides the population distribution of diffusion coefficients (‘diffusion spectrum') for the POI (magenta). Chromatin-bound histone H2B (brown) and freely diffusing nuclear localization sequence (NLS; orange) serve as controls. This analysis reveals differently diffusing subpopulations. Most TFs exhibit a chromatin-bound population and one or more diffusing subpopulations. Fast SMT also reveals whether the distribution of angles (theta) between successive displacements exhibits anisotropy, i.e. whether it is uniform in all directions (orange) or backward-biased (magenta). Slow SMT (right) can be used to measure residence times of immobile molecules, e.g. of a TF bound to chromatin.

SMT involves localizing and tracking individual fluorescently labeled molecules over time. More than a decade of advances in microscopy [13,14], genome editing [15] self-labeling tags [16], and photoswitchable fluorophores [17–20] have expanded the scope of SMT from *in vitro* mixtures [21–23] to cell membranes [24,25], to the interior of nuclei in live cells and embryos [26–40]. Several challenges had to be overcome: First, superresolution methods were developed to resolve protein molecules closer together than the diffraction limit of light, using sparse and stochastic labeling with either photoactivatable fluorophores or self-labeling tags [41,42]. Second, detecting the miniscule amount of light emitted from each fluorophore required the development of sensitive electron-multiplying charge-coupled devices (EMCCD) and complementary metal oxide semiconductor (CMOS) cameras [43–46]. Third, optical methods were developed to minimize the background fluorescence and scattered light that would otherwise overwhelm the weak fluorescence from single molecules. The most commonly used approach, highly inclined and laminated optical sheet (HILO) illumination [47], passes a laser beam obliquely through the sample, making it possible to visualize fluorophores in the cell interior. Another option, light-sheet microscopy, illuminates a single plane using a thin sheet of light and detects emission from only that plane [48–50]. A schematic of a typical SMT experiment in the mammalian cell nucleus is shown in Figure 1A.

SMT experiments fall into two broad categories: ‘Slow' SMT observes molecules bound to slow-moving scaffolds, such as chromatin [33,51–53]. A relatively long exposure time — hundreds of milliseconds to seconds — blurs out fast-moving molecules, leaving only bound molecules visible as discrete spots. Slow SMT can measure how long molecules bind chromatin, i.e. their residence time, and how they move while bound [54,55]. In contrast, ‘fast’ SMT detects both fast- and slow-moving molecules and can measure their diffusion coefficient, chromatin-bound fraction, and angular anisotropy [40,56–58]. Even with brief exposure times of 10 ms or less, fast-moving molecules appear blurred, but this motion blur can be reduced by pulsing the excitation laser like a strobe light for only 1–2 ms per frame [27]. Notably, trajectories are much shorter in fast SMT than in slow SMT due to faster photobleaching at the higher laser power used for fast SMT. (Figure 1B,C)

The first step in SMT data analysis is to localize and track single molecules, which can be done using various algorithms [59–61]. In slow SMT, a residence time distribution is obtained by classifying ‘bound' molecules that move less than a certain distance and tabulating how many frames they persist. Interpreting this distribution is complicated by the fact that fluorophores may disappear not only due to dissociation but also due to photobleaching, defocalization, or tracking errors [62]. This can be corrected for by comparison to a control protein, such as histone H2B, that remains bound to chromatin on the timescale of the experiment [36], or by pausing illumination for various time intervals between frames [39,63,64]. While residence time distributions are conventionally fit to single- or multi-exponential models to determine dissociation rate constants for discrete states [36,39,64], others have proposed that a power law distribution provides a better model of dissociation from binding sites with a range of affinities [55]. An alternative method infers distributions of dissociation rates using a numerical inverse Laplace transform [65].

For fast SMT, fitting the mean squared displacement (MSD) as a function of time provides a simple way to estimate diffusion coefficients. However, this approach suffers from systematic bias when applied to three-dimensional samples, because faster-moving molecules are more rapidly lost to defocalization. This bias can be corrected mathematically [36,39,56]. Spot-On, an analysis algorithm from our group, fits displacement histograms to infer the diffusion coefficient, localization error, and relative abundance of two or three distinct populations of molecules, such as those that are freely diffusing or chromatin bound [56]. Bayesian analysis approaches have also been developed that can infer the number of discrete diffusive states, along with their diffusion coefficients and transition rates [66,67]. An approach recently developed in our lab relies on Bayesian inference of posterior probabilities over a large two-dimensional array of states with different values of diffusion coefficient and localization error. Discrete populations are distinguishable as peaks in the resulting probability distribution [68–70].

## TF dynamics: what has SMT revealed about transcription?

SMT has enabled a new form of live-cell biochemistry, illuminating previously unseen behaviors of transcription regulators. For instance, it is tempting when gazing at chromatin immunoprecipitation sequencing (ChIP-seq) peaks to imagine that proteins form stable complexes on chromatin. However, SMT has shown that this is generally not the case. Residence time measurements by slow SMT have revealed that different transcription regulators interact with chromatin over a wide range of timescales, from milliseconds to minutes [33,37,39,55,64,71]. For example, residence times of GTFs in yeast and mammalian cells ranged from under 10 s to slightly over a minute [72–74]. These results, and others, have been collected in a database (www.mir-lab.com/dynamics-database). It should be noted that the accuracy of residence time measurements depends sensitively on correction for photobleaching and defocalization of fluorophores, and it will be important to validate the consistency of these measurements across multiple labs.

Fast SMT measurements can reveal how the chromatin bound fraction of a protein changes in response to genetic or chemical perturbations, providing a powerful way to dissect the biochemistry of protein–DNA interactions in live cells. In the case of Type I nuclear receptors, fast SMT revealed an increase in chromatin binding upon ligand treatment [52,63,69,75,76]. Combining SMT with chemical genetics in yeast revealed the *in vivo* order of assembly of GTFs and Pol II into the PIC [74].

Fast SMT has also allowed us to directly visualize how TFs locate their binding sites. The classic ‘facilitated search' model of von Hippel and Berg proposes that DNA-binding proteins find specific sites by alternating between three-dimensional diffusion and one-dimensional sliding along DNA [77]. While single-molecule studies *in vitro* have directly observed 1D sliding by TFs, this would be difficult to resolve in live-cell SMT experiments [27,78,79]. However, SMT has revealed rapid transitions between mobile and immobile states of TFs, and analysis of TF mutants suggests that most immobile molecules associate nonspecifically with chromatin [33,39,80,81], consistent with a rapidly alternating 1D and 3D search.

Another intriguing feature of target search has emerged from analyzing angles between successive displacements. The motion of the transcriptional activator c-Myc is well described by regular Brownian motion, in which these displacement angles have a uniform (isotropic) distribution [40]. In contrast, fast SMT of the pause-release factor P-TEFb and DNA-binding protein CTCF revealed a highly anisotropic, backward-biased angular distribution [40,57]. Anisotropic diffusion of CTCF depends on a putative RNA binding domain, suggesting that interactions with RNA transiently trap CTCF in small zones. Like 1D sliding along DNA, anisotropic diffusion could in theory increase the rate at which a protein binds target sites by reducing the effective dimensionality of the space that it explores. Modeling of CTCF binding and diffusion, for instance, suggested that anisotropic movement could increase its on-rate for specific sites by ∼2.5-fold [57]. However, the mechanisms underlying anisotropic motion and its functional consequences remain to be further explored.

SMT also provides a way to detect weak interactions between proteins that are otherwise difficult to study, such as those mediated by intrinsically disordered regions (IDRs). Particularly enriched in TF activation domains, IDRs mediate weak, flexible, and multivalent interactions between many types of proteins [82–84]. IDRs can profoundly influence protein behavior. A recent study from our lab revealed that it is differences in the IDR, not the DNA-binding domain, that dictate the chromatin-bound fraction of different paralogs of the HIFα transcription factor [70]. Weak, multivalent interactions can also drive liquid–liquid phase separation, which is often invoked to explain formation of nuclear sub-compartments that regulate transcription. SMT can help discern if this occurs in specific cases. SMT experiments have shown that various proteins diffuse more slowly inside nucleoli than outside, as predicted for a distinct, higher-viscosity liquid phase [68,85]. In contrast, enrichment of Pol II in herpesvirus replication compartments (RCs) involves elevated binding to DNA with the same diffusion coefficient inside and outside RCs [86]. Conversely, depletion of Pol II molecules from inactive X chromosomes involved reduced chromatin binding, with no change in diffusion coefficient, arguing against physical exclusion from a distinct condensed phase [87]. RCs, inactive X chromosomes, and nucleoli have the experimental advantage that they are relatively large. Given that the localization error of moving molecules is typically tens of nanometers, new methods will be required to discern the material properties of smaller, sub-diffraction-limited protein assemblies, such as those that form at enhancers.

## Pushing the envelope: learning more from SMT

**Figure 2.**
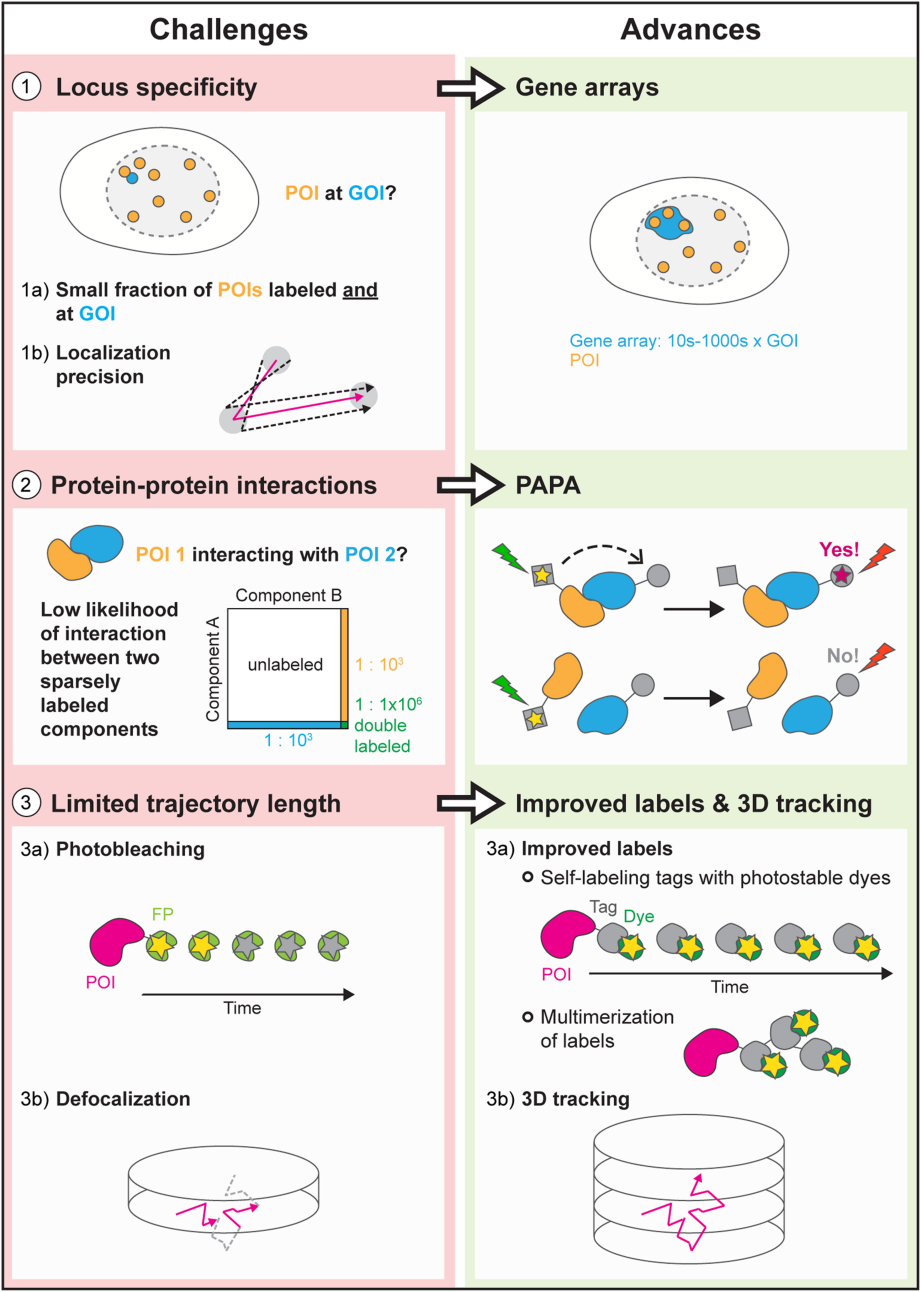
Challenges and advances in live-cell SMT. Current challenges in live-cell SMT, with a focus on transcription regulation, and selected recent advances in overcoming each problem. POI, protein of interest. GOI, gene of interest. PAPA, proximity-assisted photoactivation.

Although SMT experiments are data-rich, there is much more that we would like to discern. We can determine what fraction of a given transcription regulator binds chromatin and measure its residence time, but we don't know where on the genome it binds. We can resolve populations of TFs with different diffusion coefficients, but this does not reveal what those complexes are biochemically. We would like to monitor transitions between protein complexes, yet photobleaching and camera frame rates limit how fast and how long we can track individual molecules. Molecules move three-dimensionally in the nucleus, but we typically track them in two dimensions. Fortunately, technical advances and new approaches provide opportunities to overcome many of these limitations (Figure 2).

### Locus specificity-where are the TFs binding?

To understand nuclear processes like transcription, it is crucial to measure the binding of proteins to the specific genomic loci where these processes occur. While various tools exist for locus labeling — *lacO* and *tetO* arrays, ParB-*parS* (also referred to as ‘ANCHOR’), and dCas9 [88–96], monitoring locus-specific binding of proteins remains challenging. One problem is statistical: SMT typically entails labeling only a small fraction of molecules, and only a small fraction of those bind a given locus, yielding small sample sizes. The second problem is that localization precision is limited, especially for moving molecules. This means that a molecule that colocalizes with a labeled locus might actually be bound to a nearby segment of chromatin within the localization error.

#### Gene array imaging

One solution is to image arrays of multiple gene copies. This increases the number of localizations detectable at the specific target while spatially separating those localizations from molecules bound to the rest of the genome. Locus-specific binding of single proteins has been visualized on *Drosophila* polytene chromosomes, naturally occurring gene arrays consisting of bundles of aligned, endo-replicated sister chromatids [97]. Naturally occurring arrays in yeast have also been used to monitor TF binding to specific target genes [53]. Slow SMT of glucocorticoid receptors (GR) in mammalian cells revealed a longer residence time and lower mobility within an array of GR-responsive genes [32,51]. Slow SMT was used to measure IDR-mediated interactions between proteins in solution and proteins bound to a large array of *lacO* sites [98]. A drawback of gene arrays in mammalian cells is that they are synthetic, and arrayed genes could in principle behave differently from naturally occurring single-copy genes.

### Protein–protein interactions: resolving complexes

In some cases, PPIs can be inferred from SMT data by analyzing the effect of chemical or genetic perturbations. However, these perturbations may have unanticipated effects on the conformational and binding equilibria of proteins, possibly resulting in inaccurate interpretation of results. It would therefore be advantageous to detect PPIs directly.

#### Two-color SMT

For some proteins, such as cell surface receptors imaged by total internal reflection fluorescence (TIRF) [99–101], PPIs can be inferred by labeling the interacting partners with different fluorophores and observing whether they colocalize and move in tandem. However, this approach only works if the proteins of interest (POIs) are so dilute that single molecules are detectable even when all molecules are labeled. As discussed above, SMT more often relies on sparse labeling, making it unlikely that both interacting partners within a single complex are labeled. Moreover, it is difficult to quantify precisely what fraction of each protein is labeled. Thus, detecting complexes by two-color SMT is neither efficient nor quantitative in most cases. A similar problem limits the use of single-molecule Förster resonance energy transfer (smFRET) as a PPI sensor in live cells [69].

#### Bimolecular fluorescence complementation (BiFC)

PPIs can also be detected by fusing the two interacting partners to two halves of a split fluorescent protein or HaloTag, which are reconstituted into a complete tag when the fusion proteins come together [102,103]. While this approach has been applied to single-molecule imaging [104–106], a major disadvantage is that it irreversibly locks together the two binding partners [102,104,107], making it incapable of accurately measuring dynamic interactions.

#### Proximity-assisted photoactivation (PAPA)

We recently reported a new method to detect PPIs by SMT [69]. While imaging a multimeric protein labeled with two different Janelia Fluor (JF) dyes, we noticed that exciting JF549 causes the reappearance of JFX650 molecules that had apparently been lost to photobleaching. Because this phenomenon requires that the two dyes be close together, we dubbed it ‘proximity-assisted photoactivation' (PAPA).

Many fluorescent dyes can undergo chemical reactions when excited, which render them non-fluorescent or ‘dark'. Dark fluorophores can reactivate either spontaneously or through direct reactivation (DR) by near-UV light. Additionally, excitation of one cyanine (Cy) dye (e.g. Cy3) can reactivate a second nearby Cy dye (e.g. Cy5) [108]. Cy dye photoswitching enabled early stochastic optical reconstruction microscopy (STORM) imaging [13], yet its application as a proximity sensor was limited by its shorter distance range than FRET (<2 nm). Additionally, Cy dyes are cell impermeable, and their photoswitching requires an oxygen scavenging system and high thiol concentrations, precluding applications to live-cell imaging.

Although the mechanism of PAPA between JF dyes is unclear, it offers distinct advantages as a proximity sensor. Unlike Cy dye photoswitching, it occurs in live cells with membrane-permeable dyes, and it is more permissive of large inter-dye separations than either Cy dye photoswitching or FRET [69]. Proof-of-concept experiments showed that PAPA can detect PPIs, both in ensemble and single-molecule fluorescence imaging, and that it can be combined with SMT to characterize specific protein complexes [69]. While much work remains to understand PAPA and to refine PAPA-SMT, this approach provides a valuable foot in the door for investigating interactions of single protein molecules in live cells.

### Limited trajectory length: monitoring transitions

Tracking a single protein molecule throughout its lifetime, from synthesis to degradation, would reveal how fast it enters and exits the nucleus, how its diffusion changes over time due to association with binding partners, and how often it binds and dissociates from chromatin. Unfortunately, tracking a single molecule for an extended period of time is difficult, particularly in fast SMT, where trajectories of diffusing molecules last no more than a few camera frames [56]. While analysis methods have been devised to glean useful information from short trajectories, collecting longer trajectories would help to resolve diffusion coefficients, residence times, and transition rates between different complexes or conformations.

Two technical limitations stand in the way: First, fluorophores photobleach rapidly at the laser powers required for SMT. Approaches to overcome photobleaching include (1) using more stable fluorophores, (2) labeling each protein with multiple fluorophores, and (3) using a structured illumination approach called MINFLUX. Second, while molecules move three-dimensionally in the cell, SMT typically images a single two-dimensional focal plane, and molecules that exit this plane can no longer be tracked [39,56].

#### Improved labels

Fluorescent proteins (FPs) transformed microscopy by making it possible to label target proteins using simple molecular cloning. Synthetic organic fluorophores, although much brighter and more photostable than FPs, have historically required chemical conjugation to target proteins, mostly restricting their use to *in vitro* experiments. The development of genetically encoded, self-labeling tags like Halo and SNAP, together with cell-permeable fluorophores like JF dyes, has combined the best of both worlds, making it possible to label target proteins with bright, photostable synthetic fluorophores in live cells [109–114]. These tools continue to be improved [115,116].

#### Multimerization of labels

Labeling a protein with multiple fluorophores increases the total fluorescence signal and allows it to be tracked longer before it is lost to photobleaching. A simple option is to fuse multiple tandem copies of HaloTag to the target protein [117]. The SunTag and MoonTag comprise 24 tandem copies of short epitopes bound by different single-chain antibodies fused to a FP [118,119]. ArrayG consists of arrays of nanobodies that bind wild-type green fluorescent protein (GFP) and enhance its brightness ∼26-fold [120]. Exchange of photobleached GFP with newly synthesized GFP enables long-term slow tracking of single proteins. A disadvantage of these large tags, especially for fast tracking, is that they can greatly slow diffusion of the target protein and impact its function [118].

#### MINFLUX

MINFLUX localizes one fluorophore at a time by continuously re-centering a ‘doughnut' beam to find the point of minimum excitation [121]. Fewer photons are required for precise localization than in widefield imaging, reducing the rate of photobleaching and enabling much longer tracking. While so far mostly applied to fixed samples, it can also be used to track moving molecules for several seconds at sampling rates of 100s of kHz [121,122]. Though more technically complex than widefield imaging and currently able to track only one molecule at a time, MINFLUX will enable applications that require long, high-resolution trajectories.

#### Three-dimensional tracking

Loss of trajectories due to defocalization could be prevented by imaging and tracking in 3D. While light-sheet microscopy can be used for 3D slow SMT [37], its volumetric imaging rate is typically too slow to track 3D diffusion of molecules. One solution is a form of multifocus microscopy (MFM) that uses specialized diffractive optics to image several *z*-planes simultaneously on a camera [33,112,123]. While MFM decreases the signal per molecule by splitting light over multiple focal planes, more efficient diffraction grating designs and brighter labels are making this less of a problem [124,125].

## Glossary

- Angular anisotropy: Deviation of the angles of successive single-molecule displacements from the uniform distribution characteristic of regular Brownian motion.
- Brownian motion: An idealized random walk in which the subsequent position of an object is drawn from a Gaussian distribution centered on its current position.
- Defocalization: Movement of objects out of the focal plane.
- Diffusion coefficient: A measure of how fast a molecule diffuses. More precisely, the constant of proportionality between its MSD and 2*nt*, where *t* is time and *n* is the number of dimensions. E.g. in two dimensions, <*r*^2^> = 4*Dt*.
- Direct reactivation (DR): Reactivation of a fluorophore from a dark state in response to absorption of UV or near-UV light.
- Highly inclined and laminated optical sheet (HILO): A type of illumination similar to TIRF in which a laser beam is allowed to propagate obliquely through a sample, illuminating the region closest to the coverslip. Unlike TIRF, the beam propagates through the sample rather than undergoing total internal reflection, permitting excitation of fluorophores slightly farther from the coverslip (e.g. in the cell nucleus).
- Intrinsically disordered regions (IDRs): protein sequences that do not adopt a fixed three-dimensional fold. IDRs have been dubbed the ‘dark matter' of the proteome—extremely common, yet difficult to characterize using the standard tools of biochemistry and structural biology.
- Localization error: Uncertainty in the spatial localization of a molecule, arising from a finite number of emitted photons, molecular motion, camera noise, etc.
- Mean-squared displacement (MSD): the mean of the squared displacement from the starting position of a random walk. For regular Brownian motion, MSD increases linearly with time.
- MINFLUX: A method in which fluorophores are localized and tracked by actively re-centering a ‘doughnut' beam on the fluorophore. This is accomplished by fast electronic feedback, which locates the beam position that gives a minimal flux of emitted photons.
- Photobleaching: destruction of fluorophores by chemical reactions that occur in the excited state.
- Proximity-assisted photoactivation (PAPA): Reactivation of one fluorophore (e.g. JFX650) from a dark state upon excitation of a second nearby fluorophore (e.g. JF549).
- Residence time: How long a molecule remains bound to its target (e.g. chromatin).
- Single-molecule tracking (SMT): a method that monitors the motions of individual fluorescently labeled molecules under a microscope. SMT is a type of single-particle tracking (SPT), which includes the tracking of objects larger than a single molecule, although in practice the terms SMT and SPT are often used interchangeably.
  o Slow SMT: Tracking molecules at a relatively slow frame rate (hundreds of milliseconds to seconds) to monitor how long molecules remain bound to a slow-moving scaffold (e.g. chromatin) and how they move while bound.
  o Fast SMT: Tracking molecules with a relatively fast frame rate (typically ≤ 10 ms) to measure their diffusion. Short 1–2 ms stroboscopic excitation pulses can also be used to reduce motion blur.
- Total internal reflection fluorescence (TIRF): A type of illumination in which light is reflected off the interface between the coverslip and the sample at an angle greater than the critical angle. The resulting total internal reflection produces an exponentially decaying evanescent field that excites fluorophores within a few hundred nanometers of the interface.

## Perspectives

- Single-molecule tracking (SMT) probes interactions of biomolecules in live cells.
- Key observables of SMT include diffusion rate and anisotropy, chromatin-bound fraction and residence time, subcellular localization, and proximity to other labeled molecules. SMT can distinguish subpopulations of a biomolecule based on differences in these parameters.
- New technologies are enhancing our ability to track molecules for extended periods of time and study specific protein–protein and protein–nucleic acid interactions.

## Open Access

Open access for this article was enabled through a transformative open access agreement between Portland Press and the University of California.

## Abbreviations

BiFC: Bimolecular fluorescence complementation
ChIP-seq: chromatin immunoprecipitation sequencing
CMOS: complementary metal oxide semiconductor
DR: direct reactivation
EMCCD: electron-multiplying charge-coupled device
FP: fluorescent protein
GFP: green fluorescent protein
GTFs: general transcription factors
HILO: highly inclined and laminated optical sheet
IDRs: intrinsically disordered regions
JF: Janelia Fluor
MFM: multifocus microscopy
MSD: mean squared displacement
PAPA: proximity-assisted photoactivation
PIC: pre-initiation complex
POI: protein of interest
Pol II: RNA polymerase II
PPIs: protein–protein interactions
RC: replication compartment
smFRET: single-molecule Förster resonance energy transfer
SMT: single-molecule tracking
STORM: stochastic optical reconstruction microscopy
TFs: transcription factors
TIRF: total internal reflection fluorescence
